# Prevalence of occult nodal metastases in squamous cell carcinoma of the temporal bone: a systematic review and meta-analysis

**DOI:** 10.1007/s00405-022-07399-3

**Published:** 2022-05-13

**Authors:** Daniele Borsetto, Ananth Vijendren, Giovanni Franchin, Neil Donnelly, Patrick Axon, Matthew Smith, Liam Masterson, Manohar Bance, Athanasios Saratziotis, Jerry Polesel, Paolo Boscolo-Rizzo, James Tysome

**Affiliations:** 1 Department of ENT, Addenbrookes Hospital, Cambridge University Hospitals NHS Foundation Trust, Cambridge, UK; 2 University of Cambridge, Cambridge, UK; 3 Department of ENT, Lister Hospital, East and North Herts NHS Trust, Stevenage, UK; 4 Department of Radiation Oncology, Centro di Riferimento Oncologico di Aviano (CRO) IRCCS, Aviano, Italy; 5 Department of Otolaryngology, Head and Neck Surgery, University Hospital of Larissa, 41110 Larissa, Greece; 6 Unit of Cancer Epidemiology, Centro di Riferimento Oncologico di Aviano (CRO) IRCCS, Aviano, Italy; 7 Department of Medical, Surgical and Health Sciences, Section of Otolaryngology, University of Trieste, Trieste, Italy

**Keywords:** Lymph node metastases, Squamous cell carcinoma, Elective neck dissection, Meta-analysis, Temporal bone carcinoma

## Abstract

**Purpose:**

Primary: To determine the rate of occult cervical metastases in primary temporal bone squamous cell carcinomas (TBSSC). Secondary: to perform a subgroup meta-analysis of the risk of occult metastases based on the clinical stage of the tumour and its risk based on corresponding levels of the neck.

**Methods:**

A systematic review and meta-analysis of papers searched through Medline, Cochrane, Embase, Scopus and Web of Science up to November 2021 to determine the pooled rate of occult lymph node/parotid metastases. Quality assessment of the included studies was assessed through the Newcastle–Ottawa scale.

**Results:**

Overall, 13 out of 3301 screened studies met the inclusion criteria, for a total of 1120 patients of which 550 had TBSCC. Out of the 267 patients who underwent a neck dissection, 33 had positive lymph nodes giving a pooled rate of occult metastases of 14% (95% CI 10–19%). Occult metastases rate varied according to Modified Pittsburg staging system, being 0% (0–16%) among 12 pT1, 7% (2–20%) among 43 pT2 cases, 21% (11–38%) among 45 pT3, and 18% (11–27%) among 102 pT4 cases. Data available showed that most of the positive nodes were in Level II.

**Conclusion:**

The rate of occult cervical metastases in TBSCC increases with pathological T category with majority of nodal disease found in level II of the neck.

**Supplementary Information:**

The online version contains supplementary material available at 10.1007/s00405-022-07399-3.

## Introduction

Temporal bone cancers account for around 0.2% of head and neck malignancies and squamous cell carcinoma (SCC) represents the commonest histopathological subtype [1].

Despite its low incidence, temporal bone squamous cell carcinomas (TBSCC) are aggressive diseases with poor survival outcomes and high morbidity. Patients usually present with a troublesome discharging ear, hearing loss, pain, head and neck lumps and not infrequently a facial palsy [2]. A combination of biopsies and CT as well as MRI imaging is essential to investigate, diagnose and stage the disease for treatment options. Although there is no Union for International Cancer Control (UICC) or American Joint Committee on Cancer Control (AJCC) classification systems for TBSCC, the modified Pittsburgh staging is commonly employed [3].

TBSCC tend to spread locally rather than metastasize to regional lymph nodes or distant sites, making radical resection of the primary lesion the mainstay of treatment. Therefore, while neck dissections (ND) are generally advocated in the presence of nodal disease, although survival of patients with neck metastases is very poor [4], its role in a clinically N0 neck (cN0) has been a subject of debate, mainly due to the lack of evidence and because many institutions will routinely treat at least the upper neck as well as the primary site with adjuvant radiotherapy. The UK national multidisciplinary guidelines on the management of head and neck cancer [5, 6] have recommended that all TBSCC with cN0 undergo neck dissection of levels 2–5 based on Rinaldo et al.'s narrative review, which estimated that the risk of occult metastases lies between 17 and 25% [7]. Others may decide on the extent of neck dissection based on the clinical staging, or on performing no neck dissection given that radiotherapy may be planned post-operatively to the neck.

With these discrepancies in mind, the aim of this systematic review and meta-analysis is to estimate the rate of occult cervical metastases in primary TBSSC and to analyse the evidence on the indication and extent of elective neck dissection in these tumours.

## Methods

### Ethical consideration

As the meta-analyses did not involve any direct patient care within or outside the NHS, Research Ethics Committee approval was not necessary. The project did not constitute a form of audit nor research and hence local audit and research department approval were not required either.

### Outcome measures

The primary outcome of this meta-analysis is the prevalence of occult cervical metastases in primary TBSSC. The secondary outcome is a) to perform a subgroup meta-analysis of the risk of occult metastases based on the clinical stage of the tumour and b) the rate of occult metastases in each level in the neck.

### Search strategy

This systematic review and meta-analysis were conducted following the preferred reporting items for systematic reviews and meta-analyses (PRISMA) checklist [8]. Medline (via Ovid), Cochrane, Embase (via Ovid), Web of Science (Core Collection) and Scopus were searched from inception through to November 2021 (Appendix 1). The research was conducted according to PRISMA criteria. A combination of MeSH terms and free-text words were utilized to search for “temporal bone squamous cell carcinoma” or “temporal bone” or “squamous cell carcinoma” AND “Neck dissection” OR “Elective” OR “Node” OR “Lymphnode” OR “Lymph node” OR “N0”.

**Fig. 1:**
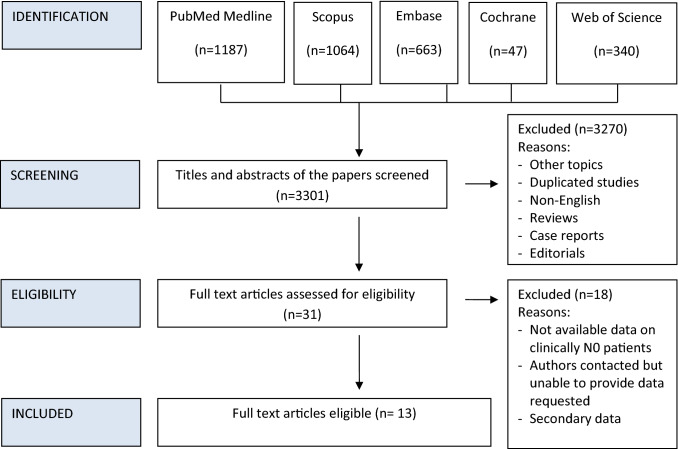
PRISMA flowchart of study inclusion process

The reference lists of articles included in this review as well as narrative reviews published in the last 10 years were also manually searched to minimize the risk of missing data. Two authors (DB and AV) independently screened all titles and abstracts generated by the search and then evaluated the full texts of all the relevant articles identified against the inclusion criteria (Fig. 1); a third author (PBR) settled discordances when present.

### Selection criteria

Studies were included in the analysis if they met the following criteria:

- Population study including previously untreated primary squamous cell carcinoma involving the temporal bone.
- Neck staging carried out by performing ultrasound (US) and/or computerized tomography (CT) and/or magnetic resonance imaging (MRI) and/or positron emission tomography (PET).
- Studies reporting complete or extractable data on the number of patients with cN0 TBSSCs, number of elective neck dissections and cases of occult metastases.

The exclusion criteria were:

- Studies that did not declare precisely how they staged the neck (palpation or imaging) before elective dissection.
- Studies with fewer than five patients.
- Non-English language studies.
- Reviews, editorials and letters.
- Studies containing aggregated and non-extractable data, or duplicated data from previously published work.
- Non-squamous cell carcinoma or metastasis.
- Recurrent disease.

We defined occult metastases (pN + /cN0) as lateral cervical lymph node metastases identified upon elective neck dissection of patients with clinically uninvolved cervical lymph nodes (cN0) at preoperative staging.

### Data extraction and statistical analysis

An electronic data-collection form was used to extract the following data from each of the included studies: author, year of publication, study design, country and period of conduction, number of patients, demographic characteristics, staging, grading of the tumours, type of imaging used to stage the neck, criteria used for elective neck dissection and dissected levels if specified, number of cN0 patients who underwent elective neck dissection and cases of occult metastases identified (pN + /cN0). The authors of the selected studies were contacted in order to gather missing information about individual patient data and attempt to perform subgroup meta-analysis. Two authors (DB, AV) independently assessed the quality of the included studies with the Newcastle–Ottawa Scale [9].

The pooled proportion of occult metastases and corresponding 95% confidence interval (CI) were calculated according to random-effects models of DerSimonian and Laird [10], using the logit transformation and weighting through the inverse variance method. Statistical heterogeneity among studies was evaluated using the *I*^2^ and *t* statistics. Influence analysis was performed when pooled proportions were estimated from five or more studies: pooled proportion was calculated by omitting one study at a time. Publication bias was assessed through a funnel plot [11].

The results of the meta-analysis were presented graphically using forest plots, plotting the individual paper, pooled proportions and corresponding 95% CI. Analyses were conducted using R 3.6, and statistical significance was claimed for *p* < 0.05 (two sided).

## Results

### Literature search

The literature review yielded 3301 articles. From the review of the titles and abstracts, 3270 articles were excluded for being irrelevant to the topic, non-English language, non-original, case reports, editorials or duplicated studies (Fig. 1). Twenty-six full-text articles were examined reporting elective neck dissection in the therapeutic management of cN0 in squamous cell carcinoma of the temporal bone. Of these, 18 were excluded as they did not meet the inclusion criteria, or because there were no available data on clinically N0 patients, the data were secondary, or because the authors were contacted but were unable to provide the data requested (authors of the studies were contacted where required for information about individual patient data to ensure a subgroup meta-analysis could be performed for specific tumour histology of squamous cell carcinoma). From the articles included in the systematic review, it was determined that 13 studies fully satisfied the inclusion criteria.

### Demographics

The 13 articles [4, 12–23] that satisfied the criteria for inclusion in the systematic review and meta-analysis were retrospective case series published between 2009 and 2021. All the studies focused on management of squamous cell carcinoma of the temporal bone. Patient demographics were only available in six studies [4, 12–15, 18]. The total population of the included studies was 1120 patients, 550 of whom had squamous cell carcinoma. Studies ranged from 6 to 124 patients with an overall median of 45.8 patients. Gender was reported in ten articles, with 239 (54%) male and 204 (46%) female.

### Core data

Data on positive SCC histology were available for all 550 patients; of which 267 patients had undergone a neck dissection. Positive metastatic lymph nodes were found in 33 of these 267 patients (Table 1). Only four studies reported the level of the positive lymph nodes [13, 14, 18, 21]. Komune et al., Ng et al., Gidley et al. and Cristalli et al. had reported a total of 55 cases undergoing prophylactic neck dissections of which eight patients had positive nodal involvement. Six of these patients were found to have nodes in level II of the neck with the levels not reported in the remaining two patients [13, 14, 18]. The data on tumour grading based on Modified Pittsburgh classification were available in 202 out of the 267 patients with the following percentages: *T*1 = 5.9%, *T*2 = 21.3%, *T*3 = 22.2%, and *T*4 = 50.5%.

**Table 1:** Description of included studies

Author and year	Patients with TBSCC	M/F ratio	Mean age	cN0 Patients who received ND	pN + patients	Positive levels	pT of cN0 patients	cN0 resulted pN +
Cristalli et al., 2009	17	15/2	74	8	1	II	*T*2:2/*T*3:5/*T*4:1	1 *T*3
Chang et al., 2009	10	9/1	56.5	2	1	na	*T*2:1/*T*4:1	1 *T*4
Zanoletti et al., 2010	47	Na	na	40	3	na	*T*1:4/*T*2:11/*T*3:13/*T*4:12	3 *T*4
Gidley et al., 2010	124	66/58	60	19	3	II	*T*1:2/*T*2:7/*T*3:1/*T*4:9	1 *T*2/ 1 *T*3/ 1 *T*4
Morris et al., 2012	39	Na	na	16	2	na	na	na
Masterson et al., 2014	60	31/29	62.5	49	2	na	*T*2:8/*T*3:7/*T*4:34	2 *T*4
McRackan et al., 2014	60	Na	73	46	8	na	na	na
Ng et al., 2015	6	4/2	63	6	1	II	*T*3:2/*T*4:3	1 *T*3
Matoba et al., 2018	25	15/10	66.5	5	0	na	*T*4:5	0
Correia-Rodrigues et al. 2020	15	6/9	77	7	1	na	*T*2:2/*T*3:3/*T*4:2	1 *T*4
Komune et al., 2021	71	28/43	65	22	3	II, III	*T*1:3/*T*2:9/*T*3:4/*T*4:5	1 *T*2/ 1 *T*3/ 1 *T*4
Piras et al., 2021	66	37/29	63	26	4	na	*T*3:2/*T*4:24	4 *T*4
Smit et al., 2021	49	28/21	65	21	4	na	*T*1:3/*T*2:3/*T*3:9/*T*4:6	1 *T*2/ 2 *T*3/ 1 *T*4

*pT* pathological staging according to Pittsburgh classification

### Meta-analysis pN + /cN0

**Fig. 2:**
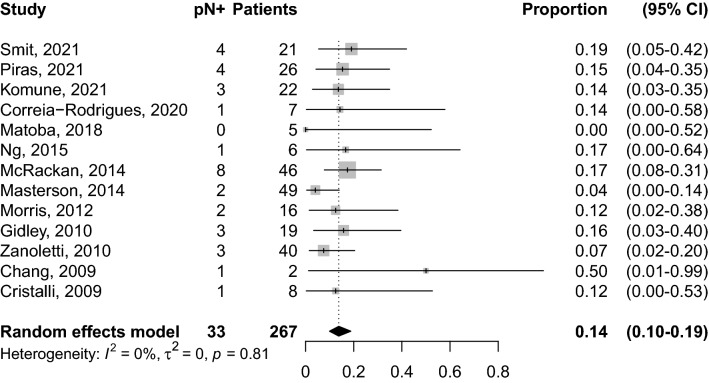
Forest plot for the prevalence of occult cervical metastases (pN+)

**Fig. 3:**
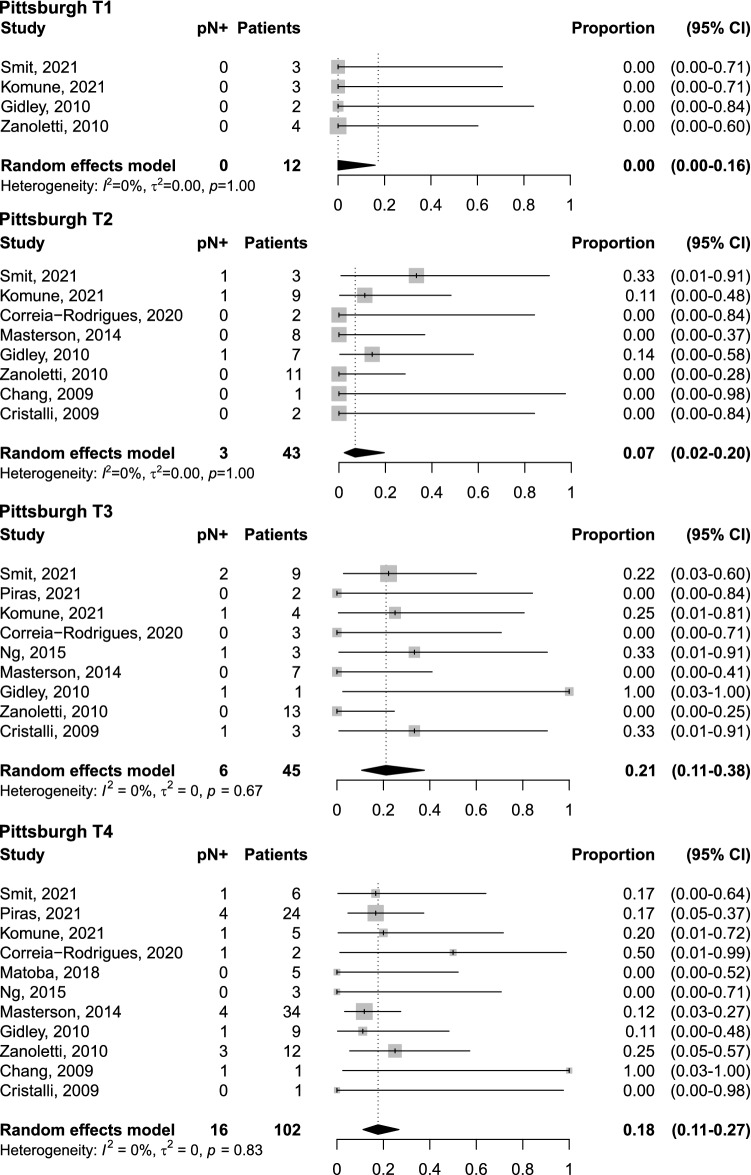
Forest plot for the prevalence of occult cervical metastases (pN+) according to Pittsburgh tumour stage

In the 13 studies, neck staging was determined by a combination of PET, MRI, US and/or CT. Of these 267 patients with TBSCC and cN0 necks, there were 33 cases were pN + /cN0, with a pooled rate of occult metastases of 14% (95% CI: 10–19%—Fig. 2); with corresponding levels of neck dissected known in 14 patients. No publication bias emerged by inspection of funnel plot (Supplementary Fig. 1.A). Influence analysis did not report substantial modification when one study was omitted at a time, with pooled proportion ranging from 13 to 15% (Supplementary Fig. 1.B). Of the 267 patients included in the meta-analysis, 202 patients had data available on the Tumour stage according to the Pittsburgh classification: 12 cases were pT1 with a pooled rate of occult metastases of 0% (95% CI: 0–16%), 43 cases were pT2 with a pooled rate of occult metastases of 7% (95% CI: 2–20%), 45 cases were pT3 with a pooled rate of occult metastases of 21% (95% CI: 11–38%), and finally 102 cases were pT4 with a pooled rate of occult metastases of 18% (CI: 11–27%) (Fig. 3).

### Quality of studies assessed and risks of biasness

Seven out of the 13 studies reported a Newcastle–Ottawa Scale (NOS) score ≥ 7, meaning they are high quality studies, but the NOS median of 7 establishes an overall low risk of bias in the meta-analysis. A detailed report on the quality of included studies according to the Newcastle–Ottawa Scale is reported in Supplementary Table 1.

## Discussion

### Summary of findings

Our meta-analyses of 267 cN0 TBSCC patients estimated an overall rate of occult lymph nodes metastases of 14% with level II being the only affected neck region, although the level was only known in 8 patients.

### Comparison to other studies

The treatment of TBSCC remains a multidisciplinary challenge. The treatment of a clinical N0 neck has been fraught with controversies and differing opinions, particularly on the benefits of a prophylactic neck dissection, the extent of dissection and the role of postoperative radiotherapy. Regional lymph node involvement has negative impact on prognosis. Morris [17] reported a 5-year disease-specific survival (DSS) of 18.8% and 80.8% in node-positive and node-negative patients while Nakagawa [24] found a 5-year estimated survival rate of 70% in patients with negative regional lymph node involvement, but a significant decline in estimated survival to 19% in patients with positive lymph node involvement. Masterson et al. [4] reported a 5-year overall survival of 0% in his cohort of TBSCC with positive lymph node involvement. The negative impact of nodal metastasis on survival supports the argument that complete surgical clearance of the tumour both at primary site and in the neck is required irrespective of the presence of nodal involvement.

Historically, elective neck dissections have been advocated for cN0 necks in patients with head and neck SCC thought to have a 20% risk of occult cervical metastases. This recommendation was based on risk–benefit analyses performed in the 1970s by authors such as Ogura et al.[25] and Lee et al.[26]. It was commonly accepted then that radical neck dissections were the main surgical approaches of choice for total disease clearance, which in effect also carries a higher morbidity with the associated removal of the accessory nerve, internal jugular vein and/or the sternocleidomastoid muscle. It is understandable why a 20% cut off was a reasonable historic choice to balance the pros and cons of the surgery. Since then, the surgical procedures for cN0 have evolved from radical neck dissections to functional, selective and highly selective procedures with consequent reduction in morbidity. These more selective approaches have been shown to adequately remove pathology while minimising morbidity such as shoulder dysfunction and have become the more mainstay form of prophylactic treatment of cervical disease. With the change in surgical technique over the years, it seems reasonable to re-evaluating the 20% cut off point and accept a lower risk of metastases as an indication for a selective neck dissection to achieve adequate disease removal and pathological neck staging.

### Clinical applicability

While previous studies have included different approaches to selective neck dissection, the little data available has only showed occult metastases at level II. As no level V found metastases were found and given the increased risk of damage to the accessory nerve, these data do not support inclusion of level V in a selective ND for a cN0 neck. While some authors have suggested a that frozen section samples are sent for Level II prior to proceeding to dissect other levels in the neck based on the results, it seems reasonable to perform a supraomohyoid or level II and III neck dissection in cN0 necks given that this also facilitates vessel preparation for a microvascular free flap as is often required in these cases [27, 28].

Based on our meta-analysis’s findings of an 14% occult risk of TBSCC cervical metastases, specifically 21% for pT3 and 18% for pT4 tumours predominantly confined to level II, we would advocate a selective level II and III neck dissection in T3 and T4 TBSCC patients after taking into account the low morbidity of the procedure and the aggressiveness of the cancer. This approach can be beneficial in a number of ways: by removing the cervical lymph nodes, one would be able to accurately stage the neck of and remove metastases not apparent on clinical staging, potentially avoiding adjuvant treatment completely if histological outcomes are favourable for this option [28]. Elective neck dissection has proved to improve the prognosis in head and neck cancers patient [29] as only a single modality treatment of the neck is required if pathologically N0, avoiding adjuvant radiotherapy of the regional lymph nodes and its related complications [30]. The low rate of occult metastases found in our pT1 (0%) and pT2 (7%) analysis and relatively small rare occurrence of T1 and T2 TBSCC would suggest that an elective neck dissection is not required echoing the recommendations from Morris et al. [17].

The role of adjuvant prophylactic radiotherapy as well as the total dose of radiation for elective neck treatment in TBSCC patients remains debatable. Although most clinicians would agree that radical surgery should be followed by postoperative radiation therapy (PORT) in cases with adverse histopathological features (e.g. advanced tumours, multiple nodal involvement, extracapsular spread, perineural invasion, positive margins) [31, 32], the overall survival of patients with stage III–IV disease remains low despite dual modality of treatment [33, 34]. Intensity-modulated radiotherapy (IMRT) has been shown to reduce the severity of toxicity and significantly improve quality of life in head and neck cancer patients [35]. However, even treatment regimens incorporating doses of IMRT with 54–63 Gy of adjuvant radiotherapy, which is considered adequate in intermediate-risk disease management according to NCCN guidelines [36] may be excessive in clinical N0 necks of TBSCC patients. As there is a general consensus that single modality of treatment should be advocated for TBSCC patients where possible, we feel that surgical excision of the TBSCC should be accompanied by a selective neck dissection after a multidisciplinary decision has been made to treat the cN0 neck.

### Strength and limitations of the study

The strength of this meta-analysis is represented by its systematic and quantitative assessment of the role of prophylactic neck dissections in TBSCC using a strict inclusion and exclusion criteria. Our limitations include the low number of cases reporting the level of metastases in the neck and the fact that the rates of occult nodal metastases reported in the literature could be underestimated as not all cN0 patients underwent a neck dissection. From a statistical point of view, this meta-analysis has the limitation of having only observational and retrospective studies included. Furthermore, there are a relative low number of cases in some of the studies predominantly as a result of the rarity of these malignancies. Further limitations of our study include:

- Insufficient of data for a statistical analysis on the prevalence of occult metastases in each of the different lymph node levels.
- Inability to determine whether selective neck dissection is better than radiotherapy.
- The inclusion criteria for many studies were not specified. This resulted in the exclusion of 17 studies in the final phase of review.

It would be helpful to perform a prospective multicentre study to compare the effectiveness of elective neck dissection and radiotherapy in TBSCC cN0 necks as data from individual units are unlikely to be sufficiently powered to influence overall management.

Finally, given the specific purposes of this investigation, the prevalence of metastases involving intra-parotid lymph nodes has not been estimated. This aspect should be investigated through a systematic review and meta-analysis specifically including studies that have addressed this issue.

## Conclusion

This meta-analysis estimated a pooled rate of occult lymph node metastases of 14%, with specific rates of 21% for pT3 tumours and 18% for pT4 tumours. Taking into account other pertinent factors such as the aim of single modality of treatment, the need to access the neck for reconstructive purposes, and the low morbidity for highly selective neck dissections, we would advocate that a selective neck dissection of at least level II or upper neck irradiation should be considered in locally advanced TBSCC.

## Supplementary Information

Below is the link to the electronic supplementary material.

Supplementary file1 (PDF 54 KB) **Supplementary Figure 1**. Funnel plot for publication bias (**A**) and influence analysis (**B**)

Supplementary file2 (DOCX 19 KB) **Supplementary Table 1**. Quality assessment of included studies according to the Newcastle-Ottawa Scale (http://www.ohri.ca/programs/clinical_epidemiology/oxford.asp)
