# Supplementary figures and images for: Prevalence of occult nodal metastases in squamous cell carcinoma of the temporal bone: a systematic review and meta-analysis
Source: Eur Arch Otorhinolaryngol. 2022 May 13;279(12):5573–81. doi: 10.1007/s00405-022-07399-3 (PMC9649468; doi:10.1007/s00405-022-07399-3)

A. Funnel plot

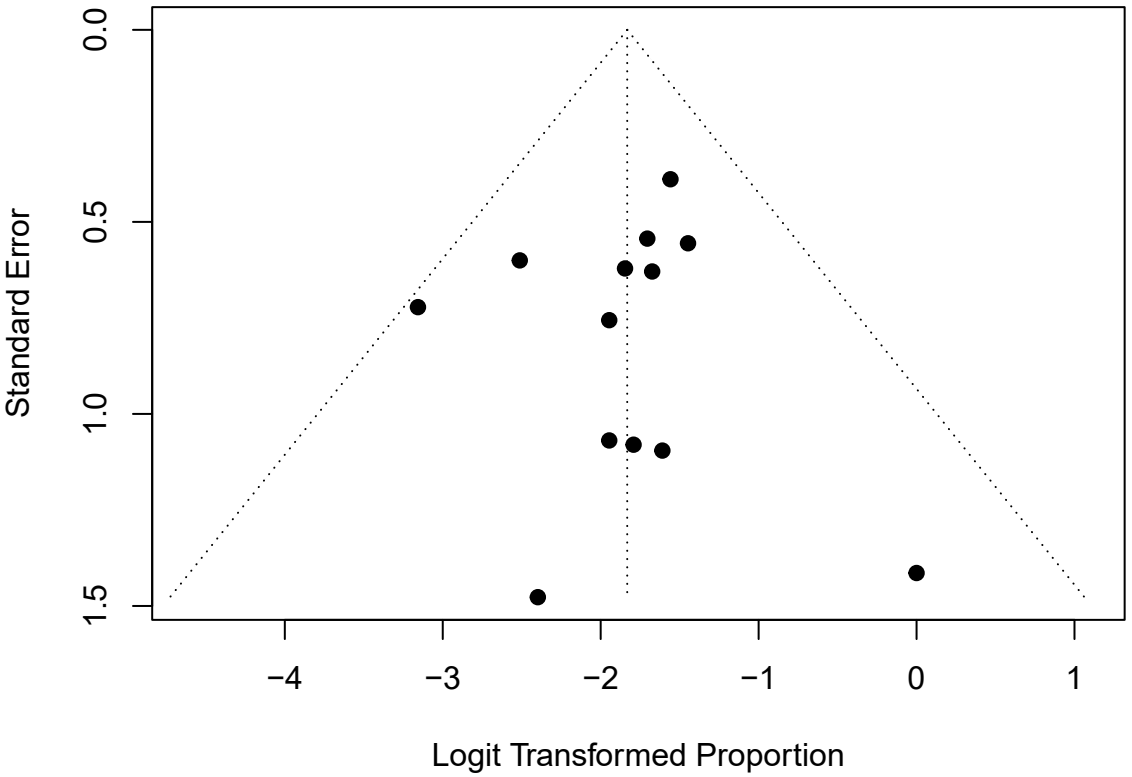

B. Sensitivity analysis

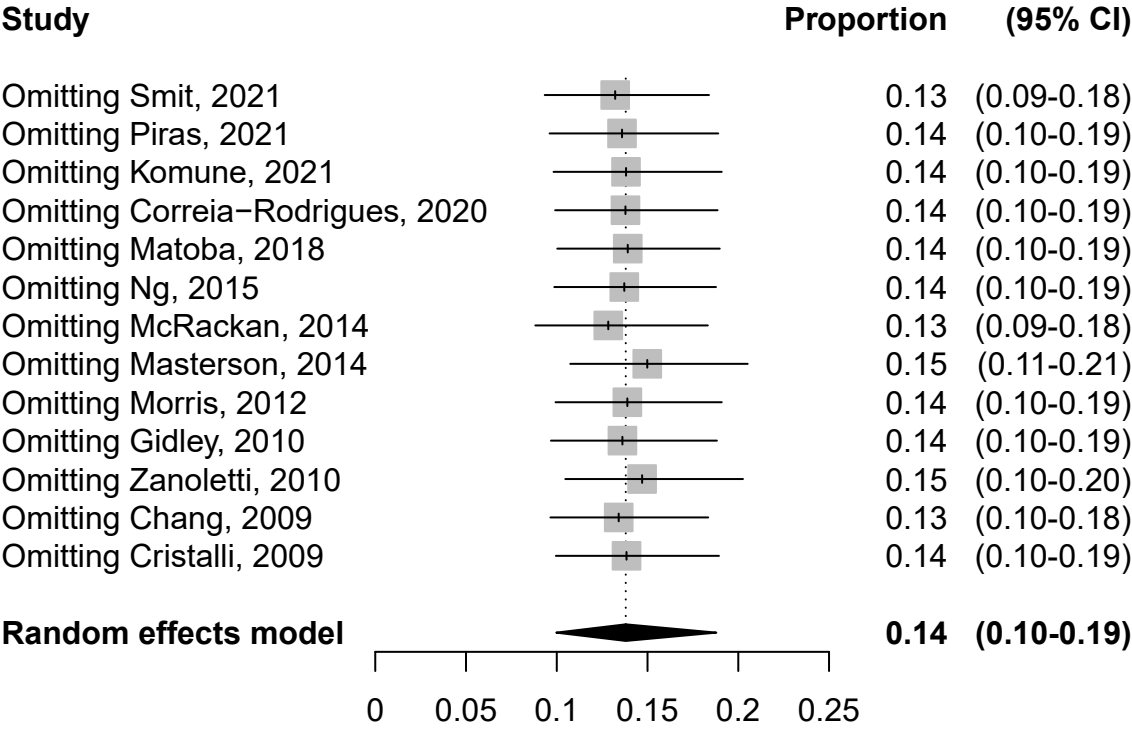

Supplement: Supplementary file 1 — Supplementary file1 (PDF 54 KB) Supplementary Figure 1. Funnel plot for publication bias (A) and influence analysis (B) [file 405_2022_7399_MOESM1_ESM.pdf]
