# Supplementary material for: Prevalence of occult nodal metastases in squamous cell carcinoma of the temporal bone: a systematic review and meta-analysis
Source: Eur Arch Otorhinolaryngol. 2022 May 13;279(12):5573–81. doi: 10.1007/s00405-022-07399-3 (PMC9649468; doi:10.1007/s00405-022-07399-3)
Supplement: Supplementary file 2 — Supplementary file2 (DOCX 19 KB) Supplementary Table 1. Quality assessment of included studies according to the Newcastle-Ottawa Scale (http://www.ohri.ca/programs/clinical_epidemiology/oxford.asp) [file 405_2022_7399_MOESM2_ESM.docx]

| Study | Selection | | | | Comparability | Outcome | | | Total |
| --- | --- | --- | --- | --- | --- | --- | --- | --- | --- |
|  | Representativeness of the exposed cohort | Selection of the non-exposed cohort | Ascertainment of exposure | Demonstration that outcome of interest was not present at start of study | Comparability of cohorts on the basis of the design or analysis controlled for confounders | Assessment of outcome | Was follow-up long enough for outcomes to occur | Adequacy of follow up of cohorts |  |
| Cristalli 2009 | * |  | * | * |  | * | * | * | 6 |
| Chang 2009 |  |  | * | * |  | * | * | * | 5 |
| Zanoletti 2010 | ** |  | * | * |  | * | * | ** | 8 |
| Gidley 2010 | ** |  | * | * |  | * | * | ** | 8 |
| Morris 2011 | ** |  | * | * |  | * | * | * | 7 |
| Masterson 2014 | ** |  | * | * |  | * | * | ** | 8 |
| McRackan 2014 | * |  | * | * |  | * |  | * | 5 |
| Ng 2015 |  |  | * | * |  | * |  | * | 4 |
| Matoba 2018 | * |  | * | * |  | * | * | * | 6 |
| Correia Rodrigues 2020 | * |  | * | * |  | * | * | * | 6 |
| Komune 2021 | ** |  | ^*^ | * |  | * | * | ** | 8 |
| Piras 2021 | ** |  | * | * |  | * | * | * | 7 |
| Smit 2021 | ** |  | * | * |  | * | * | * | 7 |
